# A new Ag^I^ complex based on 1-[(1*H*-benzimidazol-1-yl)meth­yl]-1*H*-1,2,4-triazole

**DOI:** 10.1107/S1600536811048501

**Published:** 2011-11-19

**Authors:** Yan-zhi Wang, Jun Zhang, Huai-xia Yang, Xiang-ru Meng

**Affiliations:** a Pharmacy College, Henan University of Traditional Chinese Medicine, Zhengzhou 450008, People’s Republic of China; b Department of Chemistry, Zhengzhou University, Zhengzhou 450001, People’s Republic of China

## Abstract

In the title complex, bis­{μ-1-[(1*H*-benzimidazol-1-yl)meth­yl]-1*H*-1,2,4-triazole}disilver(I) dinitrate, [Ag_2_(C_10_H_9_N_5_)_2_](NO_3_)_2_, the Ag^I^ ion is nearly linearly coordinated [N—Ag—N angle is 155.72 (14)°] by two 1-[(1*H*-benzimidazole-1-yl)meth­yl]-1*H*-1,2,4-triazole (bmt) ligands. In addition, two bmt ligands link two Ag^I^ ions, forming a dinuclear unit with an Ag⋯Ag distance of 5.0179 (15) Å. The whole complex is generated by an inversion centre. The dinuclear units and the NO_3_^−^ counter-ions are connected by N—H⋯O hydrogen bonds and weak Ag⋯O inter­actions [2.831 (5), 2.887 (5) and 2.908 (5) Å], leading to a three-dimensional structure.

## Related literature

For background to complexes based on benzimidazole or triazole and their derivatives, see: Yang *et al.* (2010 ▶); Li *et al.* (2010 ▶); Tian *et al.* (2011 ▶); Zhang *et al.* (2011 ▶). 
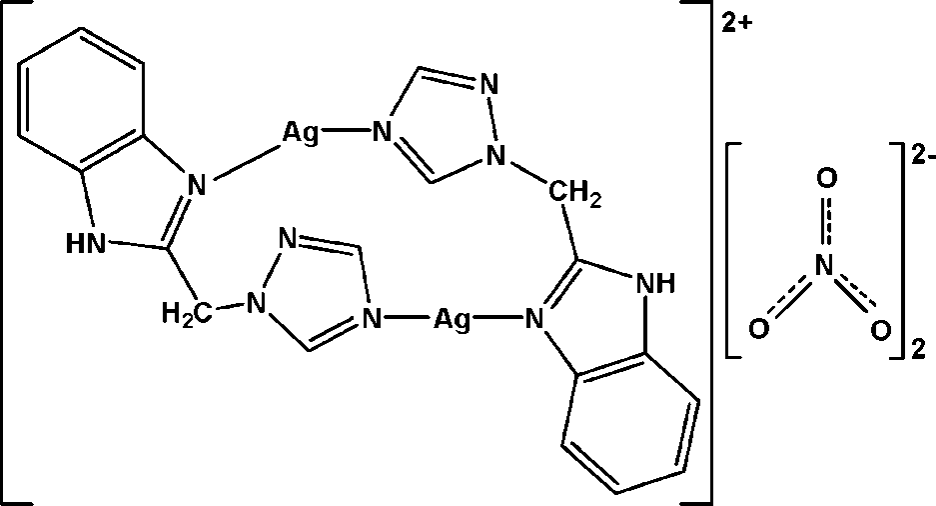


## Experimental

### 

#### Crystal data

[Ag_2_(C_10_H_9_N_5_)_2_](NO_3_)_2_
*M* *_r_* = 738.20
Monoclinic, 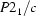

*a* = 9.4947 (19) Å
*b* = 13.569 (3) Å
*c* = 10.174 (2) Å
β = 114.56 (3)°
*V* = 1192.1 (4) Å^3^
*Z* = 2
Mo *K*α radiation
μ = 1.71 mm^−1^
*T* = 293 K
0.19 × 0.17 × 0.14 mm

#### Data collection

Rigaku Saturn diffractometer
Absorption correction: multi-scan (*CrystalClear*; Rigaku/MSC, 2006 ▶) *T*_min_ = 0.737, *T*_max_ = 0.796
9572 measured reflections
2158 independent reflections
1952 reflections with *I* > 2σ(*I*)
*R*_int_ = 0.034

#### Refinement

*R*[*F* ^2^ > 2σ(*F* ^2^)] = 0.043
*wR*(*F* ^2^) = 0.095
*S* = 1.09
2158 reflections
181 parameters
H-atom parameters constrained
Δρ_max_ = 0.70 e Å^−3^
Δρ_min_ = −0.28 e Å^−3^

### 

Data collection: *CrystalClear* (Rigaku/MSC, 2006 ▶); cell refinement: *CrystalClear*; data reduction: *CrystalClear*; program(s) used to solve structure: *SHELXS97* (Sheldrick, 2008 ▶); program(s) used to refine structure: *SHELXL97* (Sheldrick, 2008 ▶); molecular graphics: *SHELXTL* (Sheldrick, 2008 ▶); software used to prepare material for publication: *SHELXTL*.

## Supplementary Material

Crystal structure: contains datablock(s) global, I. DOI: 10.1107/S1600536811048501/vm2136sup1.cif

Structure factors: contains datablock(s) I. DOI: 10.1107/S1600536811048501/vm2136Isup2.hkl

Additional supplementary materials:  crystallographic information; 3D view; checkCIF report

## Figures and Tables

**Table 1:** Hydrogen-bond geometry (Å, °)

*D*—H⋯*A*	*D*—H	H⋯*A*	*D*⋯*A*	*D*—H⋯*A*
N2—H2*B*⋯O2	0.86	2.08	2.849 (6)	148

**Fig. 1.**
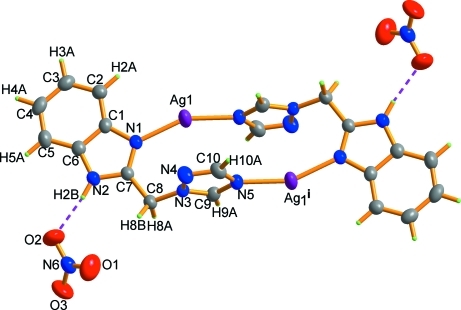
View of the title complex showing labeling and 30% probability displacement ellipsoids. Hydrogen bonds are indicated by dashed lines. Symmetry code: (i) -x - 1, -y + 2, -z.

## supplementary crystallographic information

### Comment

Many complexes based on benzimidazole or triazole and their derivatives have been synthesized and characterized owing to the strong coordination abilities of these multidentate N-heterocyclic ligands and the interesting properties and potential applications of these complexes (Yang *et al.*, 2010; Li *et al.*, 2010; Tian *et al.*, 2011; Zhang *et al.*, 2011). We are engaged in the synthesis of unsymmetrical N-heterocyclic ligands and have synthesized the compound 1-[(1*H*-benzimidazole-1-yl)methyl]-1*H*-1,2,4-triazole (bmt). In this work, we selected this compound as ligand and generated a new complex [Ag_2_(C_10_H_9_N_5_)_2_](NO_3_)_2_, (I), which is reported here.

In complex (I) each Ag^I^ ion is two-coordinated by two N atom from one triazole group and one benzimidazole group of two different 1-[(1*H*-benzimidazole-1-yl)methyl]-1*H*-1,2,4-triazole ligands and the nitrate anion does not coordinate to the Ag^I^ ion (Fig. 1). Two bmt ligands bridge two Ag^I^ ions leading to a dinuclear unit [Ag_2_(C_10_H_9_N_5_)_2_] with Ag1—Ag1^i^ distance of 5.0179 (15) Å (symmetry code: (i) *-x*-1, *-y*+2, *-z*). [Ag_2_(C_10_H_9_N_5_)_2_] units and NO_3_^-^ groups are linked through weak Ag···O interactions and N—H···O hydrogen bonds (Table 1) resulting in a three-dimensional packing in solid state.

### Experimental

The ligand 1-[(1*H*-benzimidazole-1-yl)methyl]-1*H*-1,2,4-triazole (0.1 mmol) in methanol (4 ml) was added dropwise to an aqueous solution (3 ml) of AgNO_3_ (0.1 mmol). The resulting solution was allowed to stand at room temperature in the dark. After four weeks good quality colorless crystals were obtained from the filtrate and dried in air.

### Refinement

H atoms are positioned geometrically and refined as riding atoms, with C-H = 0.93 (aromatic) and 0.97 (CH_2_) Å and N-H = 0.86 Å and with U_iso_(H) = 1.2 U_eq_(C,N).

### Crystal data

**Table:**

[Ag_2_(C_10_H_9_N_5_)_2_](NO_3_)_2_	*F*(000) = 728
*M**_r_* = 738.20	*D*_x_ = 2.057 Mg m^−^^3^
Monoclinic, *P*2_1_/*c*	Mo *K*α radiation, λ = 0.71073 Å
Hall symbol: -P 2ybc	Cell parameters from 3140 reflections
*a* = 9.4947 (19) Å	θ = 2.4–27.9°
*b* = 13.569 (3) Å	µ = 1.71 mm^−^^1^
*c* = 10.174 (2) Å	*T* = 293 K
β = 114.56 (3)°	Prism, colourless
*V* = 1192.1 (4) Å^3^	0.19 × 0.17 × 0.14 mm
*Z* = 2	

### Data collection

**Table:**

Rigaku Saturn diffractometer	2158 independent reflections
Radiation source: fine-focus sealed tube	1952 reflections with *I* > 2σ(*I*)
graphite	*R*_int_ = 0.034
Detector resolution: 28.5714 pixels mm^-1^	θ_max_ = 25.3°, θ_min_ = 2.4°
ω scans	*h* = −11→10
Absorption correction: multi-scan (*CrystalClear*; Rigaku/MSC, 2006)	*k* = −16→16
*T*_min_ = 0.737, *T*_max_ = 0.796	*l* = −12→11
9572 measured reflections	

### Refinement

**Table:**

Refinement on *F*^2^	Primary atom site location: structure-invariant direct methods
Least-squares matrix: full	Secondary atom site location: difference Fourier map
*R*[*F*^2^ > 2σ(*F*^2^)] = 0.043	Hydrogen site location: inferred from neighbouring sites
*wR*(*F*^2^) = 0.095	H-atom parameters constrained
*S* = 1.09	*w* = 1/[σ^2^(*F*_o_^2^) + (0.0419*P*)^2^ + 1.7006*P*] where *P* = (*F*_o_^2^ + 2*F*_c_^2^)/3
2158 reflections	(Δ/σ)_max_ = 0.001
181 parameters	Δρ_max_ = 0.70 e Å^−^^3^
0 restraints	Δρ_min_ = −0.28 e Å^−^^3^

### Special details

**Table:**

Geometry. All e.s.d.'s (except the e.s.d. in the dihedral angle between two l.s. planes) are estimated using the full covariance matrix. The cell e.s.d.'s are taken into account individually in the estimation of e.s.d.'s in distances, angles and torsion angles; correlations between e.s.d.'s in cell parameters are only used when they are defined by crystal symmetry. An approximate (isotropic) treatment of cell e.s.d.'s is used for estimating e.s.d.'s involving l.s. planes.
Refinement. Refinement of *F*^2^ against ALL reflections. The weighted *R*-factor *wR* and goodness of fit *S* are based on *F*^2^, conventional *R*-factors *R* are based on *F*, with *F* set to zero for negative *F*^2^. The threshold expression of *F*^2^ > σ(*F*^2^) is used only for calculating *R*-factors(gt) *etc*. and is not relevant to the choice of reflections for refinement. *R*-factors based on *F*^2^ are statistically about twice as large as those based on *F*, and *R*- factors based on ALL data will be even larger.

### Fractional atomic coordinates and isotropic or equivalent isotropic displacement parameters (Å^2^)

**Table:**

	*x*	*y*	*z*	*U*_iso_*/*U*_eq_	
Ag1	−0.34103 (4)	0.89095 (3)	0.20814 (4)	0.04938 (17)	
N1	−0.0927 (4)	0.9097 (2)	0.3164 (4)	0.0326 (8)	
N2	0.1479 (4)	0.9666 (3)	0.3996 (4)	0.0354 (8)	
H2B	0.2248	1.0035	0.4080	0.043*	
N3	−0.1878 (4)	1.0476 (2)	0.0747 (4)	0.0320 (8)	
N4	−0.1943 (5)	0.9813 (3)	−0.0280 (4)	0.0449 (10)	
N5	−0.4205 (4)	1.0613 (3)	−0.0931 (4)	0.0374 (8)	
N6	0.3779 (5)	1.1840 (3)	0.4722 (5)	0.0470 (10)	
O1	0.3112 (6)	1.1880 (4)	0.3398 (4)	0.0877 (14)	
O2	0.3944 (6)	1.1033 (3)	0.5326 (5)	0.0818 (14)	
O3	0.4245 (5)	1.2605 (3)	0.5397 (5)	0.0801 (13)	
C1	0.0033 (5)	0.8484 (3)	0.4281 (4)	0.0320 (9)	
C2	−0.0334 (6)	0.7641 (3)	0.4866 (5)	0.0387 (10)	
H2A	−0.1339	0.7396	0.4515	0.046*	
C3	0.0871 (6)	0.7193 (3)	0.5989 (5)	0.0446 (12)	
H3A	0.0670	0.6633	0.6409	0.054*	
C4	0.2383 (6)	0.7552 (3)	0.6518 (5)	0.0454 (12)	
H4A	0.3158	0.7223	0.7277	0.055*	
C5	0.2760 (5)	0.8380 (3)	0.5946 (5)	0.0415 (11)	
H5A	0.3769	0.8618	0.6295	0.050*	
C6	0.1542 (5)	0.8840 (3)	0.4813 (5)	0.0345 (10)	
C7	−0.0011 (5)	0.9789 (3)	0.3042 (4)	0.0306 (9)	
C8	−0.0451 (5)	1.0643 (3)	0.2027 (4)	0.0352 (10)	
H8A	−0.0574	1.1222	0.2528	0.042*	
H8B	0.0379	1.0774	0.1732	0.042*	
C9	−0.3227 (5)	1.0931 (3)	0.0341 (5)	0.0353 (10)	
H9A	−0.3452	1.1409	0.0881	0.042*	
C10	−0.3371 (6)	0.9929 (4)	−0.1265 (5)	0.0450 (11)	
H10A	−0.3772	0.9569	−0.2120	0.054*	

### Atomic displacement parameters (Å^2^)

**Table:**

	*U*^11^	*U*^22^	*U*^33^	*U*^12^	*U*^13^	*U*^23^
Ag1	0.0295 (2)	0.0625 (3)	0.0471 (3)	0.00013 (17)	0.00685 (17)	0.00857 (18)
N1	0.0290 (19)	0.0329 (19)	0.0328 (19)	0.0005 (16)	0.0098 (16)	−0.0039 (15)
N2	0.030 (2)	0.038 (2)	0.036 (2)	−0.0027 (16)	0.0114 (16)	−0.0009 (16)
N3	0.0292 (19)	0.0302 (17)	0.0335 (19)	0.0019 (15)	0.0100 (16)	0.0020 (15)
N4	0.040 (2)	0.046 (2)	0.047 (2)	0.0002 (19)	0.016 (2)	−0.0166 (19)
N5	0.030 (2)	0.041 (2)	0.036 (2)	0.0014 (17)	0.0097 (17)	0.0027 (17)
N6	0.036 (2)	0.056 (3)	0.047 (3)	−0.007 (2)	0.015 (2)	−0.004 (2)
O1	0.103 (4)	0.097 (3)	0.048 (2)	0.023 (3)	0.016 (2)	−0.003 (2)
O2	0.089 (3)	0.060 (3)	0.088 (3)	−0.008 (2)	0.029 (3)	0.028 (2)
O3	0.067 (3)	0.064 (3)	0.094 (3)	−0.025 (2)	0.019 (2)	−0.029 (2)
C1	0.033 (2)	0.032 (2)	0.028 (2)	0.0053 (18)	0.0098 (18)	−0.0064 (18)
C2	0.040 (3)	0.034 (2)	0.040 (2)	0.000 (2)	0.014 (2)	−0.0028 (19)
C3	0.060 (3)	0.033 (2)	0.037 (3)	0.004 (2)	0.018 (2)	0.000 (2)
C4	0.055 (3)	0.041 (3)	0.034 (2)	0.018 (2)	0.012 (2)	0.001 (2)
C5	0.034 (3)	0.049 (3)	0.035 (2)	0.005 (2)	0.008 (2)	−0.007 (2)
C6	0.036 (3)	0.035 (2)	0.031 (2)	0.0020 (19)	0.013 (2)	−0.0054 (18)
C7	0.030 (2)	0.031 (2)	0.028 (2)	−0.0005 (18)	0.0106 (18)	−0.0052 (17)
C8	0.031 (2)	0.034 (2)	0.036 (2)	−0.0011 (19)	0.0093 (19)	−0.0042 (19)
C9	0.037 (3)	0.037 (2)	0.032 (2)	0.003 (2)	0.015 (2)	0.0031 (19)
C10	0.040 (3)	0.049 (3)	0.043 (3)	−0.007 (2)	0.014 (2)	−0.010 (2)

### Geometric parameters (Å, °)

**Table:**

Ag1—N1	2.163 (4)	N6—O2	1.233 (5)
Ag1—N5^i^	2.171 (4)	C1—C6	1.390 (6)
N1—C7	1.321 (5)	C1—C2	1.399 (6)
N1—C1	1.397 (5)	C2—C3	1.377 (6)
N2—C7	1.353 (5)	C2—H2A	0.9300
N2—C6	1.382 (5)	C3—C4	1.395 (7)
N2—H2B	0.8600	C3—H3A	0.9300
N3—C9	1.325 (5)	C4—C5	1.380 (7)
N3—N4	1.361 (5)	C4—H4A	0.9300
N3—C8	1.454 (5)	C5—C6	1.396 (6)
N4—C10	1.318 (6)	C5—H5A	0.9300
N5—C9	1.313 (6)	C7—C8	1.492 (6)
N5—C10	1.352 (6)	C8—H8A	0.9700
N5—Ag1^i^	2.171 (4)	C8—H8B	0.9700
N6—O3	1.222 (5)	C9—H9A	0.9300
N6—O1	1.229 (5)	C10—H10A	0.9300
			
N1—Ag1—N5^i^	155.72 (14)	C4—C3—H3A	118.9
C7—N1—C1	105.5 (4)	C5—C4—C3	121.9 (4)
C7—N1—Ag1	131.1 (3)	C5—C4—H4A	119.1
C1—N1—Ag1	123.3 (3)	C3—C4—H4A	119.1
C7—N2—C6	107.6 (4)	C4—C5—C6	116.2 (4)
C7—N2—H2B	126.2	C4—C5—H5A	121.9
C6—N2—H2B	126.2	C6—C5—H5A	121.9
C9—N3—N4	109.8 (4)	N2—C6—C1	105.5 (4)
C9—N3—C8	128.8 (4)	N2—C6—C5	132.3 (4)
N4—N3—C8	121.2 (3)	C1—C6—C5	122.2 (4)
C10—N4—N3	102.1 (4)	N1—C7—N2	112.2 (4)
C9—N5—C10	103.0 (4)	N1—C7—C8	127.7 (4)
C9—N5—Ag1^i^	125.8 (3)	N2—C7—C8	120.1 (4)
C10—N5—Ag1^i^	131.1 (3)	N3—C8—C7	112.8 (3)
O3—N6—O1	118.6 (5)	N3—C8—H8A	109.0
O3—N6—O2	122.2 (5)	C7—C8—H8A	109.0
O1—N6—O2	119.1 (5)	N3—C8—H8B	109.0
C6—C1—N1	109.2 (4)	C7—C8—H8B	109.0
C6—C1—C2	121.1 (4)	H8A—C8—H8B	107.8
N1—C1—C2	129.7 (4)	N5—C9—N3	110.5 (4)
C3—C2—C1	116.5 (4)	N5—C9—H9A	124.8
C3—C2—H2A	121.8	N3—C9—H9A	124.8
C1—C2—H2A	121.8	N4—C10—N5	114.6 (4)
C2—C3—C4	122.2 (4)	N4—C10—H10A	122.7
C2—C3—H3A	118.9	N5—C10—H10A	122.7
			
N5^i^—Ag1—N1—C7	−24.9 (5)	C4—C5—C6—N2	179.8 (4)
N5^i^—Ag1—N1—C1	149.8 (3)	C4—C5—C6—C1	−0.4 (6)
C9—N3—N4—C10	0.5 (5)	C1—N1—C7—N2	0.3 (4)
C8—N3—N4—C10	−176.4 (4)	Ag1—N1—C7—N2	175.7 (3)
C7—N1—C1—C6	0.1 (4)	C1—N1—C7—C8	−178.8 (4)
Ag1—N1—C1—C6	−175.7 (3)	Ag1—N1—C7—C8	−3.3 (6)
C7—N1—C1—C2	179.6 (4)	C6—N2—C7—N1	−0.7 (5)
Ag1—N1—C1—C2	3.7 (6)	C6—N2—C7—C8	178.5 (3)
C6—C1—C2—C3	0.2 (6)	C9—N3—C8—C7	114.0 (5)
N1—C1—C2—C3	−179.2 (4)	N4—N3—C8—C7	−69.8 (5)
C1—C2—C3—C4	−0.4 (6)	N1—C7—C8—N3	−23.6 (6)
C2—C3—C4—C5	0.1 (7)	N2—C7—C8—N3	157.4 (4)
C3—C4—C5—C6	0.3 (6)	C10—N5—C9—N3	0.7 (5)
C7—N2—C6—C1	0.7 (4)	Ag1^i^—N5—C9—N3	178.7 (3)
C7—N2—C6—C5	−179.5 (4)	N4—N3—C9—N5	−0.8 (5)
N1—C1—C6—N2	−0.5 (4)	C8—N3—C9—N5	175.8 (4)
C2—C1—C6—N2	180.0 (4)	N3—N4—C10—N5	0.0 (5)
N1—C1—C6—C5	179.6 (4)	C9—N5—C10—N4	−0.4 (5)
C2—C1—C6—C5	0.1 (6)	Ag1^i^—N5—C10—N4	−178.3 (3)

Symmetry codes: (i) −*x*−1, −*y*+2, −*z*.

### Hydrogen-bond geometry (Å, °)

**Table:**

*D*—H···*A*	*D*—H	H···*A*	*D*···*A*	*D*—H···*A*
N2—H2B···O2	0.86	2.08	2.849 (6)	148.
